# Progression of Aortic Regurgitation After Subarterial Ventricular Septal Defect Repair: Optimal Timing of the Operation

**DOI:** 10.1007/s00246-019-02206-z

**Published:** 2019-09-13

**Authors:** Hanna Jung, Joon Yong Cho, Youngok Lee

**Affiliations:** Department of Thoracic and Cardiovascular Surgery, School of Medicine, Kyungpook National University, Kyungpook National University Hospital, 130 Dongdeok-ro, Jung-gu, Daegu, 41944 Republic of Korea

**Keywords:** Aortic regurgitation, Aortic valve, Aortic valve prolapse, Complications, Pediatric cardiac surgery, Ventricular septal defect

## Abstract

In patients with subarterial ventricular septal defect (VSD), the progression of aortic regurgitation (AR) still remains unclear. This review is to identify the incidence of AR progression after VSD repair and to determine the optimal operation timing for subarterial VSD repair with or without aortic valve prolapse or AR. From January 2002 to December 2015, 103 patients who underwent subarterial VSD repair alone at our hospital were reviewed. All patients routinely underwent echocardiography (echo) performed by our pediatric cardiologists. The operative approach was through the pulmonary artery in all patients. The median age of patients at operation was 10 months (range 3 to 16.5 months). Eighty-nine patients (86.4%) underwent subarterial VSD closure before the age of 4 years. In the preoperative evaluation, 27.2% (28 patients) of the patients showed more than faint degree AR. The mean follow-up duration after VSD repair was 6.6 ± 4.0 years. In the latest follow-up echo after VSD repair, four patients had more than mild degree AR owing to aortic valve abnormalities or delayed operation period. Among them, AR progression occurred in only one patient (0.98%). Early and accurate assessment of the anatomical morphology of the aortic valve and optimal operation timing may be important to achieve better outcomes after repair and to prevent the development of aortic valve complications.

## Introduction

Subarterial ventricular septal defect (VSD) is relatively common in Asians. The incidence of subarterial VSD in Asian countries accounts for approximately one-quarter of all VSD cases requiring surgical closure, which is quite rare in Western populations. Patients with subarterial VSD, due to the lack of support of the aortic valve in the absence of the infundibular septum [1], are particularly concerned about aortic valve prolapse and aortic regurgitation (AR), which eventually tend to deform the aortic valve and could possibly result in rupture of the sinus of Valsalva in adulthood [2–4].

For these reasons, surgical patch closure of the defect with or without concurrent aortic valve repair has been recommended as a gold standard for the treatment, but the ideal time for subarterial VSD repair still remains controversial. Some suggest that defects should be closed as soon as AR is detected or even earlier in the presence of aortic valve prolapse [1], whereas others advice early elective closure of the defect before the development of aortic valve complications or even earlier at the time of diagnosis of subarterial VSD [2, 5]. Perhaps, the relatively low risk of cardiopulmonary bypass needs to overcome the benefits of preventing aortic valve complications [3]. There are many reviews about aortic valve complications associated with subarterial VSD, but there are limited data about AR progression after subarterial VSD repair [6, 7].

The present study aimed to identify the incidence of AR progression after VSD repair and to determine the optimal operation timing for subarterial VSD repair with or without aortic valve prolapse or AR.

## Patients and Methods

### Patients

Between January 2002 and December 2015, 103 patients with subarterial VSD underwent corrective operation at Kyungpook National University Hospital. We retrospectively reviewed the clinical records, echocardiograms, operative findings, and surgical outcomes of all these patients.

The indications for surgery were rapid breathing and/or failure to thrive due to congestive heart failure, recurrent respiratory infection such as pneumonia or bronchitis due to excessive blood flow to the lung, or occurrence and/or progression of aortic valve complications such as aortic valve prolapse, AR, or other standard indications for VSD closure.

### Echocardiography

All patients routinely underwent echocardiography (echo) performed by our pediatric cardiologists. Preoperative echo was performed at least once before the operation to evaluate the leaflets of the aortic valve; particularly, the right coronary cusp was carefully evaluated for the prolapse. AR was diagnosed by two-dimensional and color doppler echo in parasternal long-axis view and was graded as absent, mild (AR jet reaching just beneath the aortic valve), moderate (AR jet reaching beyond the anterior cusp of the mitral valve but not reaching the left ventricular apex), or severe (AR jet reaching the left ventricular apex) [2, 4]. Postoperative echo was performed at least once before discharge or on the first out-patient clinic follow-up in the early postoperative period, and every two or three years in the late postoperative period.

### Surgical Technique

The operations were all performed by our established routine surgical method. Through the transpulmonary approach, patch closure was performed under extracorporeal circulation. Bicaval venous cannulation and the left ventricle were vented through the right superior pulmonary vein. After cross-clamping of the aorta, a cardioplegic solution was infused. At this time, the defect was exposed by vertical incision of the pulmonary artery and aortic valve prolapse was identified by visualizing through that defect.

All defects were closed with pledget-supported interrupted sutures circumferentially around the defect, including the posterior cusps of the pulmonary valve. In most of the cases, glutaraldehyde-treated autologous pericardium patches were used. The patch was designed slightly smaller than the defect to uphold the leaflets of the aortic valve. At the end of the operation primary closure of the pulmonary artery was performed.

### Statistical Analysis

Continuous variables were expressed as a median and interquartile range for non-normally distributed data and mean ± standard deviation for normally distributed data. Differences between continuous variables were assessed by the Mann–Whitney *U* test for non-normally distributed data and unpaired *t*-test for normally distributed data. Categorical variables were expressed as numbers and percentages. Differences between categorical variables were assessed using the χ^2^ test. The relationships between the grade of pre- and postoperative AR and the age at operation were analyzed by scatter plot. Values were considered to be statistically significant when the *p*-value was less than 0.05. All statistical analyses were performed using IBM SPSS version 23.0 for Windows (IBM Corp., Armonk, NY, USA).

## Results

### Patients’ Characteristics

The patients’ demographic characteristics are described in Table 1. The median age of patients at operation was 10 months (range 3 to 16.5 months). The patients’ median body weight and body surface area at the time of the operation were 9.4 kg (range 5.8 to 11.0 kg) and 0.5 m^2^ (range 0.3 to 0.6 m^2^), respectively. Their median hospital stay was 8 days (range 7 to 11 days), and postoperative hospital stay was 7 days (range 6 to 9 days). The patients had been followed for a mean 6.6 ± 4.0 years, and their mean age at the latest follow-up was 8.3 ± 5.5 years.

**Table 1:** Patients’ characteristics

Characteristics	*n* = 103
Age (month)	10 (3–16.5)
Sex	
Female	30 (29.1%)
Male	73 (70.9%)
Weight (kg)	9.4 (5.8–11.0)
Body surface area (m^2^)	0.5 (0.3–0.6)
Hospital stay (day)	8 (7–11)
Postoperative hospital stay (day)	7 (6–9)
Follow-up duration (year)	6.6 ± 4.0
Age at the latest follow-up (year)	8.3 ± 5.5

Data are median (IQR) or n (%) or mean ± standard deviation

*IQR* interquartile range

**Fig. 1:**
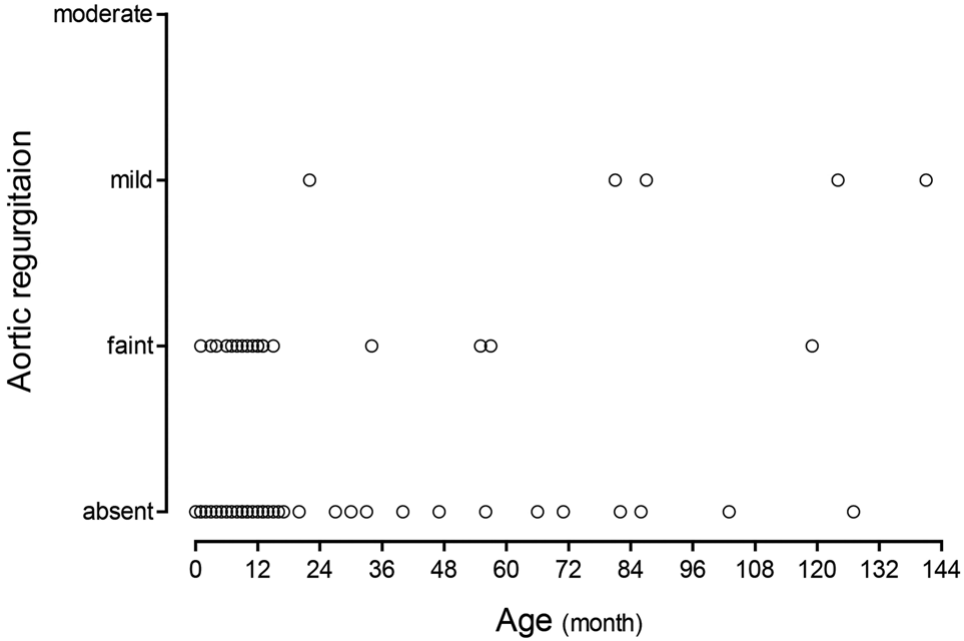
Scatter plot showing the correlation of the grade of preoperative aortic regurgitation with the age at operation

Preoperative echocardiogram data are shown in Table 2. Sixty-five patients (63.1%) had aortic valve prolapse. All patients only had right coronary cusp prolapse in this group. Twenty-eight (27.2%) patients had preoperative AR and their AR grade was all less than mild degree. None of the patients had severe AR. The mean size of the defect measured by echo was 5.1 ± 2.3 mm. The correlation of the grade of preoperative AR with the age at operation is demonstrated as a scatter plot in Fig. 1.

**Table 2:** Preoperative echocardiogram data

Characteristics	*n* = 103
Aortic valve prolapse	65 (63.1%)
Aortic regurgitation	28 (27.2%)
Absent	75
≤ Faint	23
Faint < ≤ mild	5
Mild < ≤ moderate	0
VSD diameter (mm)	5.1 ± 2.3

Data are *n* (%) or mean ± standard deviation

*VSD* ventricular septal defect

Intraoperative outcomes are listed in Table 3. The median clamp and pump times in the operation was 36 min (range 29 to 47.5 min) and 61 min (range 48 to 72 min), respectively. The median VSD diameter measured in the operative field was 5 mm (range 2 to 7 mm). Most of the patients (99%) used glutaraldehyde-treated autologous pericardial patch for VSD closure.

**Table 3:** Intraoperative outcomes

Characteristics	*n* = 103
Clamp time (min)	36 (29–48)
Pump time (min)	61 (48–72)
Cardioplegic solution infusion route	
Antegrade	102 (99%)
Retrograde	1 (1%)
VSD diameter (mm)	5 (2–7)
Patch material used	
Pericardium^a^	102 (99%)
Gore-Tex	1 (1%)
Combined procedure	
ASD closure	43 (42%)
PDA ligation	12 (12%)
MV repair	7 (7%)
TV repair	3 (3%)
RVOT muscle resection	1 (1%)

Data are *n* (%) or median (IQR)

*VSD* ventricular septal defect, *ASD* atrial septal defect, *PDA* patent ductus arteriosus, *MV* mitral valve, *TV* tricuspid valve, *RVOT* right ventricular outflow tract, *IQR* interquartile range

^a^Glutaraldehyde-treated autologous pericardium

There was no operative mortality and no deaths during the hospital stay or the follow-up period. No patients required reoperation for residual VSD.

### Progression of AR After Subarterial VSD Repair

**Fig. 2:**
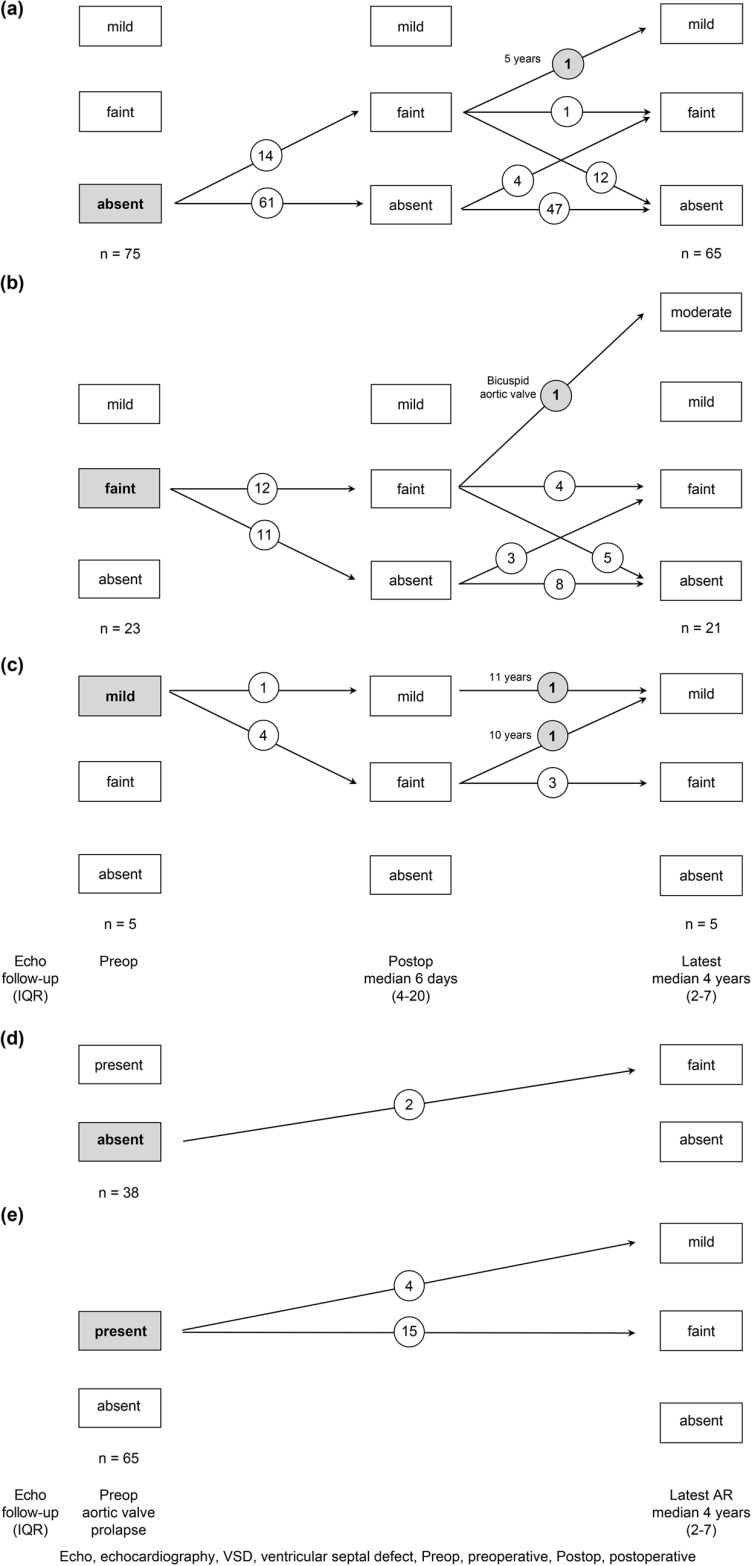
Evolution of aortic regurgitation (AR) in total 103 patients. **a** Degree of AR after subarterial ventricular septal defect (VSD) repair in 75 patients with preoperative absent AR. **b** Degree of AR after subarterial VSD repair in 23 patients with preoperative faint degree AR. **c** Degree of AR after subarterial VSD repair in 5 patients with preoperative mild degree AR. **d** Degree of AR after subarterial VSD repair in 38 patients without preoperative aortic valve prolapse. **e** Degree of AR after subarterial VSD repair in 65 patients with preoperative aortic valve prolapse

The evolution of AR in the total of 103 patients, from preoperative echo to the latest echo follow-up, is demonstrated in Fig. 2. Follow-up echo was performed immediately after the operation at a median of 6 days (range 4 to 20 days), and the latest follow-up echo was performed at a median 4 years (range 2 to 7 years).

Seventy-five patients did not have AR before the operation. Of the 28 patients who had preoperative AR, 23 and 5 patients had faint and mild degree AR, respectively. In 75 patients without AR before the operation, 14 patients developed faint degree AR postoperatively, but eventually in the latest follow-up echo, only 5 patients had faint degree AR and one patient had mild degree AR (Fig. 2a). Twenty-three patients had preoperative faint degree AR. AR disappeared in the postoperative follow-up echo in less than half of patients, and eventually AR disappeared in the latest follow-up echo in more than 75% of patients. Unfortunately, one patient had postoperative AR progression that increased into moderate degree AR (Fig. 2b). Five patients showed preoperative mild degree AR. Two of the five patients had persisting mild degree AR but it did not progress at the latest follow-up echo (Fig. 2c).

We also reviewed the association between preoperative aortic valve prolapse and progression of AR. Thirty-eight patients did not have preoperative aortic valve prolapse; of these, only 2 patients had faint degree of AR in the latest follow-up echo (Fig. 2d). Sixty-five patients (63.1%) had aortic valve prolapse before the operation; of these, 19 patients had more than a faint degree of AR in the latest follow-up echo (Fig. 2e).

### The Influence of Age at the Operation on AR Progression

**Fig. 3:**
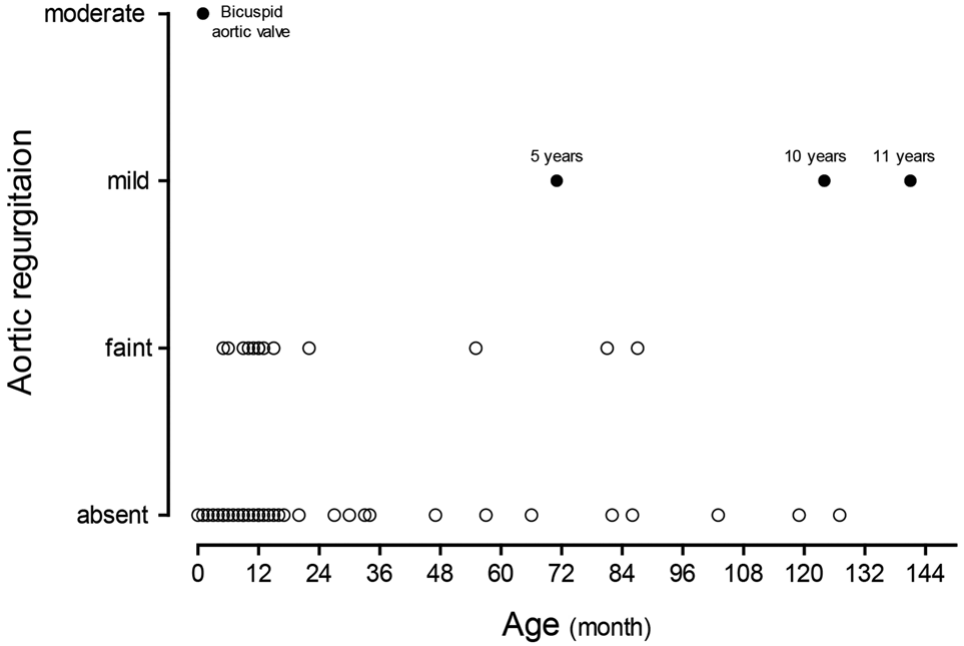
Scatter plot showing the correlation of the grade of postoperative aortic regurgitation with the age at operation

The correlation of the grade of postoperative AR with the age at operation is shown as a scatter plot in Fig. 3. Given that a gap was seen between 3 and 4 years, we divided the patients into the following groups according to their age at operation to compare the echocardiogram data and operative outcomes between the groups: 3 years old or younger (≤ 3-year group) and older than 4 years old (> 4-year group). A significant difference was shown in the latest follow-up echo between the ≤ 3-year and > 4-year groups (*p* < 0.001) described in Table 4.

**Table 4:** Comparison of echocardiogram data and operative outcomes by the age at operation

Characteristics	≤ 3-year group (*n* = 77)	> 4-year group (*n* = 13)	*p*-value
Sex			1.000
Female	24 (31.2%)	4 (30.8%)	
Male	53 (68.8%)	9 (69.2%)	
Preoperative aortic valve prolapse			0.035
Absent	33 (42.9%)	1 (7.7%)	
Present	44 (57.1%)	12 (92.3%)	
Preoperative aortic regurgitation			< 0.001
Absent	59 (76.6%)	6 (46.2%)	
≤ Faint	17 (22.1%)	3 (23.1%)	
Faint < ≤ mild	1 (1.3%)	4 (30.8%)	
VSD diameter (mm)^a^	5.0 (1.0–6.5)	5.0 (4.0–8.0)	0.633
Clamp time (min)	38.0 (29.0–48.0)	34.0 (30.0–39.0)	0.326
Pump time (min)	63.0 (48.0–74.0)	54.0 (50.0–60.0)	0.131
Hospital stay (day)	8 (7–11)	8 (7–8)	0.821
Postoperative hospital stay (day)	7 (6–9)	7 (6–7)	0.753
Latest follow-up aortic valve prolapse			1.000
Absent	74 (96.1%)	12 (92.3%)	
Present	3 (3.9%)	1 (7.7%)	
Latest follow-up aortic regurgitation			< 0.001
Absent	65 (84.4%)	7 (53.8%)	
≤ Faint	12 (15.6%)	3 (23.1%)	
Faint < ≤ mild	0 (0.0%)	3 (23.1%)	

In this comparison, one bicuspid aortic patient and 12 patients who were lost to follow-up were excluded

Data are *n* (%) or median (IQR)

*VSD* ventricular septal defect, *IQR* interquartile range

^a^Operative outcome

### Cases of Aortic Regurgitation Progression

As shown in Fig. 2, four patients had more than mild degree AR in the latest follow-up echo. Characteristics of the four patients with AR progression are shown in Table 5.

**Table 5:** Cases of aortic regurgitation progression

Case	Sex/age at operation	AR degree in preop echo	VSD size	AR degree in postop echo	AR degree in latest echo	Cause of AR progression
1	Male/1 month	Faint	10 mm	Faint postop day 6th	Moderate post op 5 year	Bicuspid aortic valve Aortic root dilatation
2	Male/5 years	Absent	1 mm	Faint postop day 39th	Mild post op 13 year	Delayed repair
3	Female/11 years	Mild	4 mm	Mild postop 1 month	Mild post op 8 year	Delayed repair
4	Female/10 years	Mild	16 mm	Faint postop 2 months	Mild post op 8 year	Delayed repair

*AR* aortic regurgitation, *preop* preoperative, *echo* echocardiography, *VSD* ventricular septal defect, *postop* postoperative

The first patient was a 1-month old boy who had faint degree AR in the preoperative echo. Given that the baby had congestive heart failure, VSD closure was performed. The bicuspid aortic valve was found in the intraoperative field, and the patient had regular out-patient clinic follow-up in the pediatric department. The latest follow-up echo was performed at 5 years postoperatively, which showed moderate degree AR with aortic root dilatation due to bicuspid aortic valve. Since the patient has no heart failure symptoms, he is still followed up in the out-patient clinic of the pediatric department. The latest chest X-ray examination showed no cardiomegaly.

The remaining patients were known to have VSD when they were born, but since they had no heart failure symptoms, their parents did not submit them to routine follow-up in the out-patient clinic of the pediatric department. VSD closure was performed when the children were 5, 11, and 10 years old. It seems that AR of the second patient gradually progressed from faint to mild degree AR. The AR of the third and fourth patients neither progressed nor improved, but persisted as mild degree AR.

## Discussion

The results of our mid-term experience show that 89 patients (86.4%) underwent subarterial VSD closure before 4 years of age. Ninety-eight patients (95.1%) had less than faint degree AR in the preoperative echo, and which improved or at least did not progress to a more severe degree postoperatively. In this study, we evaluated the incidence of AR progression after subarterial VSD repair. All patients had less than mild degree AR immediately after VSD closure. None of the patients required aortic valve repair or any other procedure during VSD closure. Excluding the bicuspid aortic valve patient, AR progression occurred in one patient (0.98%) after VSD closure, but the postoperative 13-year follow-up echo showed no more than mild degree AR, which did not require interventions.

Amano et al. [7] also reviewed 91 patients to determine the occurrence rate and predictors of AR progression after subarterial VSD repair. Their patients’ median age was 3 years (range 0 to 38 years). The incidence of late AR progression after subpulmonic infundibular VSD repair alone was 7.7%, and postoperative VSD leakage was reported as a significant risk factor. Although their outcomes were different from ours due to the different age range, they present a similar key message that aortic valve deformity may be an important risk factor for postoperative AR progression, which require early operation to prevent the development of aortic valve complications.

The complications of aortic valve are most concerned in subarterial VSD due to natural history and anatomical morphology. The lack of anatomical muscular support directly beneath the aortic valve leads to herniation of the leaflet, and the additional ‘Venturi effect’ created by the shunt flow during systole pulls the leaflet through the defect [4, 8]. Moreover, the tendency of the defect in subarterial VSD generally does not undergo spontaneous closure. Occasionally, the defect could be seen as a ‘functionally’ restrictive defect but it is actually a large defect that is covered by the prolapsing aortic valve leaflet, which could mislead or delay the treatment strategy [3].

In our retrospective review, the proportion of patients with preoperative aortic valve prolapse was 63.1% (65 patients), regardless of the presence of preoperative AR; of these, 19 patients had more than a faint degree of AR in the latest follow-up echo. In 38 patients who had neither preoperative aortic valve prolapse nor preoperative AR, only 2 patients developed more than a faint degree of AR in the latest follow-up echo. The proportion of preoperative AR was 27.2% (28 patients), which is not more than what we have expected, and the preoperative AR degree was less than mild. We presume that, in subarterial VSD patients, surgical closure should be performed as soon as possible when AR or any abnormality on aortic valve develops, even if the patient only had aortic valve prolapse or faint degree AR without heart failure symptoms.

Figure 2 and Table 5 describe the patients with mild degree AR in the latest follow-up echo. We assume that VSD closure was performed too late in these patients, and the aortic valve may have been deformed before VSD closure.

Therefore, subarterial VSD requires close monitoring from the moment of diagnosis, and early surgical closure of subarterial VSD is strongly recommended once aortic valve deformity is present and even before the onset of aortic valve deformity, as preoperative AR or aortic valve prolapse are the risk factors of postoperative residual AR or AR progression [2, 4–6, 8]. However, given that there is no definite time for elective closure of subarterial VSD, some pediatric cardiologists still delay the surgical closure and wait until an aortic valve deformity develops, which may place the child at risk for developing AR progression even after the surgical closure.

## Conclusion

AR progression occurred in only one of our patients after VSD closure, which was unexpectedly low (0.98%). Moreover, 95.1% (98 patients) of the patients had less than faint degree AR in preoperative echo, showing an unexpectedly low prevalence of preoperative AR in subarterial VSD patients who underwent surgical closure. Only the patients with aortic valve abnormalities or delayed operation had AR progression or persisting more than mild degree AR. Therefore, early and accurate assessment of anatomical morphology of the aortic valve and optimal operation timing may be important to achieve better outcomes after repair and prevent the development of aortic valve complications.

## Limitations

This study was limited owing to the use of retrospective data. The retrospective design of this study did not allow the comparison of the two groups of patients with similar degrees of aortic insufficiency, as the patients in one group were operated and the patients in the other group were only followed up conservatively for a while. A prospective study might not be appropriate owing to ethical reasons, because making the patients receiving conservative treatment to wait to see what happens to the aortic valve can be harmful to them; thus, a retrospective design is more appropriate for this study.

## References

[1] van Doorn C, de Leval MR, Stark JF, Leval MR, Tsang VT (2006). Ventricular septal defects. Surgery for congenital heart defects.

[2] Cheung Y-f, Chiu CS, Yung T-c, Chau AK (2002). Impact of preoperative aortic cusp prolapse on long-term outcome after surgical closure of subarterial ventricular septal defect. Ann Thorac Surg.

[3] Shamsuddin AM, Chen YC, Wong AR, Le TP, Anderson RH, Corno AF (2016). Surgery for doubly committed ventricular septal defects. Interact Cardiovasc Thorac Surg.

[4] Komai HMD, Naito YMD, Fujiwara KMD, Noguchi YMD, Nishimura YMD, Uemura SMD (1997). Surgical strategy for doubly committed subarterial ventricular septal defect with aortic cusp prolapse. Ann Thorac Surg.

[5] Sim EKW, Grignani RT, Wong ML, Quek SC, Wong JCL, Yip WCL, Lee CN (1999). Influence of surgery on aortic valve prolapse and aortic regurgitation in doubly committed subarterial ventricular septal defect. Am J Cardiol.

[6] Salih HG, Ismail SR, Kabbani MS, Abu-Sulaiman RM (2016). Predictors for the outcome of aortic regurgitation after cardiac surgery in patients with ventricular septal defect and aortic cusp prolapse in saudi patients. Heart Views.

[7] Amano M, Izumi C, Imamura S, Onishi N, Tamaki Y, Enomoto S, Miyake M, Tamura T, Kondo H, Kaitani K, Yamanaka K, Nakagawa Y (2016). Progression of aortic regurgitation after subpulmonic infundibular ventricular septal defect repair. Heart.

[8] Devlin PJ, Russell HM, Monge MC, Patel A, Costello JM, Spicer DE, Anderson RH, Backer CL (2014). Doubly committed and juxtaarterial ventricular septal defect: outcomes of the aortic and pulmonary valves. Ann Thorac Surg.

